# Human Paraoxonase 1 as a Pharmacologic Agent: Limitations and Perspectives

**DOI:** 10.1155/2014/854391

**Published:** 2014-10-20

**Authors:** Priyanka Bajaj, Rajan K. Tripathy, Geetika Aggarwal, Abhay H. Pande

**Affiliations:** Department of Biotechnology, National Institute of Pharmaceutical Education and Research (NIPER), Sector 67, Sahibzada Ajit Singh Nagar, Punjab 160062, India

## Abstract

Human PON1 (h-PON1) is a multifaceted enzyme and can hydrolyze (and inactivate) a wide range of substrates. The enzyme shows anti-inflammatory, antioxidative, antiatherogenic, ant-diabetic, antimicrobial, and organophosphate (OP)-detoxifying properties. However, there are certain limitations regarding large-scale production and use of h-PON1 as a therapeutic candidate. These include difficulties in producing recombinant h-PON1 (rh-PON1) using microbial expression system, low hydrolytic activity of wild-type h-PON1 towards certain substrates, and low storage stability of the purified enzyme. This review summarizes the work done in our laboratory to address these limitations. Our results show that (a) optimized polynucleotide sequence encoding rh-PON1 can express the protein in an active form in *E. coli* and can be used to generate variant of the enzyme having enhanced hydrolytic activity, (b) *in vitro* refolding of rh-PON1 enzyme can dramatically increase the yield of an active enzyme, (c) common excipients can be used to stabilize purified rh-PON1 enzyme when stored under different storage conditions, and (d) variants of rh-PON1 enzyme impart significant protection against OP-poisoning in human blood (*ex vivo*) and mouse (*in vivo*) model of OP-poisoning. The rh-PON1 variants and their process of production discussed here will help to develop h-PON1 as a therapeutic candidate.

## 1. Introduction

Human paraoxonase 1 (h-PON1) (EC 3.1.8.1) is a ~43 kDa polypeptide of 355 amino acids [1, 2]. It is primarily synthesized in the liver and is secreted into the bloodstream where it is associated with a category of high density lipoprotein particles [3–6]. The h-PON1 is a multitasking enzyme and can hydrolyze different types of substrates. Various hydrolytic activities of h-PON1 can be broadly grouped into three categories: arylesterase, phosphotriesterase, and lactonase [7]. The enzyme exhibits anti-inflammatory, antioxidative, antiatherogenic, antidiabetic, antimicrobial, and OP-neutralizing properties [1, 8–12]. Recent reports suggest that h-PON1 also plays an important role in the metabolism of certain drugs [13–15].

## 2. Protective Role of PON1 and PON1 as a Potential Therapeutic Candidate

The level and the activity of serum PON1 in individuals suffering from cardiovascular diseases, liver diseases, diabetes, renal diseases, cancer, and obesity are considerably lower than in the normal subjects [5, 16–21]. The level and the activity of circulating PON1 are also considered as novel biomarkers for the evaluation of these diseases in humans [22–26]. Animals deficient in PON1 have been found to be more susceptible to these disease conditions and the overexpression of h-PON1 or administration of purified PON1 in these animals has been shown to prevent/retard the development of these disease conditions [10, 12, 27–30]. The beneficial role of h-PON1 in OP-poisoning is also well demonstrated. Animals deficient in PON1 (knockout animals) have been found to be more susceptible to OP-poisoning compared to their wild-type counterpart and administration of purified PON1 has been shown to provide protection against OP-poisoning in various animal models [12, 31–39]. In some cases, PON1 has been shown to provide better protection than the existing antidotes of OP-poisoning [40]. Antimicrobial role of PON1 is also well documented in the literature [8, 41, 42]. Thus, h-PON1 has emerged as a strong candidate for the development of therapeutic intervention against a variety of conditions in humans [8, 12, 43–55].

## 3. Problems Associated with the Development of h-PON1 as a Therapeutic Candidate

Native h-PON1 does not have sufficiently high hydrolytic activity against all its substrates; therefore, there is a need to develop improved variant(s) of h-PON1 having enhanced hydrolytic activity against desired substrate(s) [45, 56, 57]. This can be done by protein engineering in which changing the amino acid residue at a particular position in h-PON1 can develop a variant of the enzyme having enhanced hydrolytic activity. To do this, a simple system for the expression and the production of rh-PON1 is urgently needed.* E. coli* expression system is the most preferred system for the manufacture of recombinant proteins [58–60]. This system also permits easy genetic manipulation to generate desired variant(s) of the target recombinant protein, characterization of which can also help in elucidating the mechanism of action of the target protein.

The production of rh-PON1 in high yield and in functionally active form using an* E. coli* expression system has been difficult until now [27, 36, 61, 62]. Numerous complex approaches were used earlier to generate active rh-PON1 with high purity and high yield using this expression system (e.g., generation of gene family reshuffled chimeric-PON1 (Chi-PON1) [27], addition of >5 extra amino acids to the recombinant enzyme [63], and use of specialized* E. coli* cells which contain additional “helper” plasmid(s) [36, 63]). These approaches resulted in either considerable alteration in the original amino acid sequence of the h-PON1 or significantly low yield of the recombinant protein.

## 4. Polymorphism in PON1 and Generation of Improved Variants

The crystal structure of h-PON1 has not been solved yet and the molecular details of how the enzyme hydrolyzes different types of substrates are also not clear. Literature suggests that amino acid residues at positions 115 and 192 in h-PON1 play an important role in modulating the hydrolytic activities of the enzyme [56, 64, 65]. In native h-PON1, histidine (H) residue occupies 115 and 134 positions while glutamine (Q) or arginine (R) is present at position 192. It is proposed that H115 forms a catalytic dyad with H134 and participates in the hydrolytic activity of PON1. Presence of Q/R at position 192 of PON1 dramatically affects the hydrolytic properties of the enzyme towards particular substrate(s). Alloform of h-PON1 containing R at position 192 efficiently degrades paraoxon while alloform carrying Q at the same position possesses better hydrolytic activity towards soman and sarin [9, 66–68]. Interestingly, PON1 from rabbit plasma contains lysine (K) residue at position 192 and exhibits very high hydrolytic activity towards paraoxon and lactones [69]. Recently, it was observed that substitution of H115 with tryptophan (W) residue increases the hydrolytic activity of the enzyme towards OP-compounds and decreases the lactone- and arylester- hydrolyzing activities of the enzyme [27, 56, 65, 70]. Based on this information and in order to understand how the enzyme hydrolyzes different types of substrates, we have generated and characterized the following variants of rh-PON1_(wt)_: rh-PON1_(H115W)_, rh-PON1_(H115W;R192K)_, rh-PON1_(H115W;R192Q)_, rh-PON1_(H115W;H134R)_, rh-PON1_(H115W;H134R;R192K)_, and rh-PON1_(L69G;S111T;H115W;H134R;R192K;F222S;T332S)_ [71, 72].

## 5. Expression of Active rh-PON1 Enzymes in* E. coli*

In order to express rh-PON1 enzymes in the active form in* E. coli*, codon optimized genes encoding rh-PON1 enzymes were generated and expressed in* E. coli*. rh-PON1 enzymes were expressed in soluble and active form and as (His)_6_-tagged proteins. These recombinant enzymes are referred as “soluble,” to differentiate them from the refolded enzymes described later in this report. The recombinant proteins were purified to homogeneity using a two-step chromatographic procedure [71, 72]. Using this procedure, we were able to get a yield of 0.2–0.25 mg of pure and active rh-PON1/g wet cell mass of* E. coli*. Characterization of rh-PON1 enzymes revealed that rh-PON1_(wt)_ is similar to native h-PON1 in terms of its hydrolytic activities as well as its amino acid sequence [71, 72].

Although we managed to express rh-PON1 enzymes in active form in* E. coli*, the final yield of the purified protein was very low despite growing the cells expressing rh-PON1 enzyme at a low temperature and inducing the expression of recombinant protein by using low concentration of inducer (i.e., IPTG), the two most important parameters that promote the expression of recombinant proteins in active form in* E. coli* [73, 74].

## 6. Production of rh-PON1 Enzymes by Refolding of Inclusion Bodies (IBs)

Being a eukaryotic protein, overexpression of rh-PON1 in* E. coli* leads to aggregation of the overexpressed protein in inactive form as IBs. Thus, it is difficult to express active rh-PON1 enzyme in high amount in* E. coli* [43, 61, 62]. Also, for the purification of recombinant proteins expressed in low amount, the presence of “tag” in the recombinant protein is essential [74]. Although, this “tag” helps in easy purification of the recombinant protein (by using affinity chromatography), it may lead to complications when such tag-containing proteins are used as drugs in physiological conditions [74].

*In vitro* refolding of recombinant proteins present in inactive form in IBs to their active form has emerged as an attractive alternative over production of soluble and active recombinant proteins [75–77]. However,* in vitro* refolding of recombinant proteins is considered as a major bottleneck in protein production scheme [75–77]. We have developed a method for production of active rh-PON1 enzymes in high yield by* in vitro* refolding of IBs [78]. The (His)_6_-tag present in the rh-PON1 enzymes was removed and the rh-PON1 enzymes containing no (His)_6_-tag were overexpressed in* E. coli* as IBs. The IBs were purified and the recombinant proteins were refolded (to their active form) by diluting the denatured protein into refolding buffer. The active enzymes were isolated from the refolding mixture by ion-exchange chromatography. Dilution along with additive assisted refolding method is a widely preferred approach for industrial scale production of recombinant proteins over other methods of* in vitro* refolding [79]. Enzymatic characterization of refolded rh-PON1 enzymes indicated that the catalytic properties of the refolded enzymes were similar to their soluble counterparts. The refolded rh-PON1 enzymes have 100% amino acid sequence identity to native h-PON1, with minimal changes necessary for enhancing its hydrolytic activity, and are devoid of any “tag” or extra amino acids. By using the procedure of* in vitro* refolding and isolation of active protein, we were able to get a yield which is significantly higher than the yield of rh-PON1 reported in the literature [78].

## 7. Storage Stability of Purified rh-PON1 Enzymes

Purified h-PON1 (recombinant or isolated from human plasma) is relatively unstable and rapidly loses its enzymatic activity when stored in aqueous buffer at 25°C or 4°C [80–85]. h-PON1 is an important protein and for successful commercial applications and use, long-term storage stability of purified rh-PON1 enzyme is important. A few attempts were made earlier by researchers to increase the stability of PON1 protein [40, 85]. But in these studies, complex physiological counterparts of h-PON1 (like reconstituted HDL [40] or human phosphate binding protein (HPBP) [85]) had been used to stabilize r-PON1. However, till now no detailed studies have been carried out to find simpler pharmaceutical excipients that can ensure continued storage of purified h-PON1 under different storage conditions without loss of its enzymatic activity.

To increase their shelf life, biotechnologically important proteins are usually stored as liquid or lyophilized formulations. We have screened various excipients for their ability to stabilize rh-PON1 when stored in either aqueous solution or lyophilized form at 25°C [86]. Our results show that glycine and serine are most effective in stabilizing the enzyme when stored in aqueous buffer at 25°C, and trehalose, maltose, and BSA exerted maximum stabilization effect when the enzyme was stored in the lyophilized form at 25°C [86]. The results suggest that simpler pharmaceutical excipients can be used to stabilize purified rh-PON1 enzymes when stored for a long period of time under different storage conditions and these results can be used to develop formulation(s) of rh-PON1 enzymes for commercial use.

## 8. Prophylactic Activity of Refolded rh-PON1 Enzyme against OP-Poisoning

OP-compounds are toxic chemicals that exert their deleterious effect by inhibiting neurotransmitter-metabolizing enzymes [87, 88]. Current treatments available for OP-poisoning are considered as unsatisfactory and inadequate, and there is an urgent need for the development of more effective treatment for OP-poisoning [44–49]. h-PON1 is a strong candidate for the development of prophylactic and therapeutic agent against OP-poisoning in humans [44–49].

Prophylactic activity of refolded rh-PON1 enzyme was studied using mouse model of OP-poisoning [11, 40]. Our results show that the refolded rh-PON1 enzyme was not toxic and was safely tolerated by the animals and pretreatment with refolded rh-PON1 enzyme imparted protection against OP-poisoning in mice [78].

## 9. Conclusion

H-PON1 is a versatile protein and possesses multiple beneficial properties. It is a potential candidate for the development of therapeutic intervention against OP-poisoning and other disease conditions in humans. Availability of a procedure to produce rh-PON1 enzymes in pure form and high yield by using microbial expression system will help tremendously in generating variants of h-PON1, characterization of which will increase our knowledge about the catalytic mechanism of the enzyme. This will also help in producing desired variant(s) of h-PON1 enzyme in large quantity so that the therapeutic potential of such variant(s) can be tested in various animal models. This will certainly help in developing h-PON1 as a pharmacologic agent in future.
